# Knowledge and risk perception towards Lassa fever infection among residents of affected communities in Ebonyi State, Nigeria: implications for risk communication

**DOI:** 10.1186/s12889-020-8299-3

**Published:** 2020-02-12

**Authors:** Ifeoma Sophia Usuwa, Christian Obasi Akpa, Chukwuma David Umeokonkwo, MaryJoy Umoke, Chukwuemeka Steve Oguanuo, Abdulhakeem Abayomi Olorukooba, Eniola Bamgboye, Muhammad Shakir Balogun

**Affiliations:** 1 Nigeria Field Epidemiology and Laboratory Training Programme (NFELTP), 50 Haile Selassie Street, Abuja, Nigeria; 2 Department of Family Medicine, University of Uyo Teaching Hospital Uyo, Uyo, Akwa Ibom State Nigeria; 3 Department of Community Medicine, Alex Ekwueme Federal University Teaching Hospital, Abakaliki, Ebonyi State Nigeria; 4 Ebonyi State, Ministry of Health (SMOH), Abakaliki, Ebonyi State Nigeria; 5 Nigeria Centre for Disease Control (NCDC), Abuja, Nigeria; 6 Department of Community Medicine, Ahmadu Bello University, Zaria, Nigeria; 7 Department of Epidemiology and Medical Statistics, University of Ibadan, Ibadan, Oyo State Nigeria; 8 African Field Epidemiology Network (AFENET), Abuja, Nigeria

**Keywords:** Knowledge, Perception, Lassa fever, Outbreak response, Communities, Health belief model, Risk communication

## Abstract

**Background:**

Lassa fever (LF) is an epidemic-prone zoonotic disease prevalent in Nigeria and Ebonyi State is a high burden area in Nigeria. Low risk perceptions have been reported to prevent appropriate preventive behaviours. We investigated the knowledge and risk perception of residents towards LF and determined the factors influencing their risk perception in communities that have reported confirmed cases of LF.

**Methods:**

We conducted a cross-sectional study in the affected wards in Abakaliki Local Government Area (LGA). We interviewed 356 adult respondents recruited across 6 settlements in 3 of the affected wards through multistage sampling technique. Information on participants’ knowledge of LF, their risk perception using the health belief model as well as factors influencing risk perception were obtained. We estimated the proportions of respondents with good knowledge and high risk perceptions. We also explored the relationship between risk perception, knowledge and sociodemographic characteristics using Chi Square and logistic regression at 5% level of significance.

**Results:**

The mean age of the participants was 33.3 ± 12.2 years, 208 (63.2%) were females, 230 (69.9%) were married and 104 (31.6%) had attained tertiary education. Though 99.1% were aware of LF infection, 50.3% among them had poor knowledge of LF symptoms and risk factors, 92.9% had high risk perception of severity, 72.4% had a high feeling of susceptibility towards LF infection, 82.5% had a high perceived self-efficacy towards LF infection, 63.5% had a low perceived benefit of LF preventive practices and 31.8% had high perceived barrier towards LF preventive practices. Good knowledge of LF was the only significant factor influencing risk perception; perceived severity: (COR: 3.0, 95%CI: 1.2–7.8), perceived susceptibility (AOR: 2.0, 95%CI: 1.25–3.3) and perceived benefit (COR: 2.1, 95%CI: 1.3–3.3).

**Conclusions:**

Good knowledge of LF influences risk perception towards LF which has great import on LF preventive practices. A gap exists in the content and acceptance of LF risk communication information in the LGA. There is a need to review the risk communication messages in the state towards LF in the community with special focus on the males and younger population.

## Introduction

Lassa fever (LF) is an epidemic-prone zoonotic viral haemorrhagic disease caused by Lassa fever virus (LFV), a member of the *Arenaviridae* family [1]. *Mastomys natalensis*, a multimammate, peri-domestic rat is the reservoir. LF is endemic in Nigeria, Liberia, Sierra Leone, Benin, Ghana, Guinea and Mali [1]. It has also been reported in Europe and North America [2]. LF affects both sexes and all age groups [1]. About 300,000–500,000 cases and 5000 deaths occur yearly in West Africa [3]. The case fatality of Lassa fever varies greatly depending on the context ranging from 1 to 2% overall, 15–20% in hospitalized patients, about 50% during epidemics and 80% during third trimester pregnancy [1]. The case fatality rates of 3–42% has been reported in Nigeria [4]. The incidence of LF is usually highest during the dry season (November–April) with outbreaks occurring often during this period [4].

Transmission of Lassa fever is mainly from rodent-to-human. Human to human transmission has been reported [1, 5]. There is currently no vaccine available for Lassa Fever, hence prevention is focused on interrupting the chain of transmission [1]. The Nigeria Centre for Disease Control (NCDC) recommends a number of measures the general public could take such as ensuring personal and environmental hygiene, practicing frequent hand washing at all times, ensuring food items are stored in rat proof containers to limit rodent to human interaction and early reporting of symptomatic cases to the treatment centre [4]. Thus, the success of outbreak prevention and control is dependent on human behavior (whether people at risk comply with behavioural recommendations) [6]. This is because misperceptions can hinder response efforts and promote further spread of the disease as human behaviour is based on attitudes, belief systems, opinions and awareness of a disease [7].

The Health Belief Model (HBM) postulates that when an individual perceives that he/she is at risk of contracting a serious disease (threat), he/she will initiate a certain health behavior to prevent it. However, this health behavior will not be adopted unless the benefits of the behavior outweigh its barriers and consequences. Those beliefs are potentiated by triggers (cues to action), which could be internal or external [8, 9]. In addition, the model proposes that certain variables such as demographic factors, and knowledge of disease, can affect an individual’s beliefs. This could indirectly influence an individual’s behavior as well as cues to action [8, 10–12]. Self-efficacy is another important facet influencing health behavior as people are more likely to adopt a behaviour if they have high self-confidence and belief in their ability to take on that behaviour.

Though discovered fifty years ago, a lot is yet to be understood about LF including the factors influencing human behaviours towards prevention and control of the disease. Understanding the risk perception of LF in communities is a prerequisite for successful development and implementation of its prevention and control strategies especially in risk communication activities [13].

Ebonyi State is one of the three high burdened states which report frequent occurrences of LF outbreaks [14]. Abakaliki Local Government Area (LGA) in Ebonyi State had the highest proportion of confirmed LF cases during the 2018 and 2019 LF outbreaks in the State. We determined the knowledge and risk perception of residents of affected communities towards LF infection using the HBM in Abakaliki LGA during a LF outbreak in the state.

## Materials and methods

### Study area and design

**Fig. 1:**
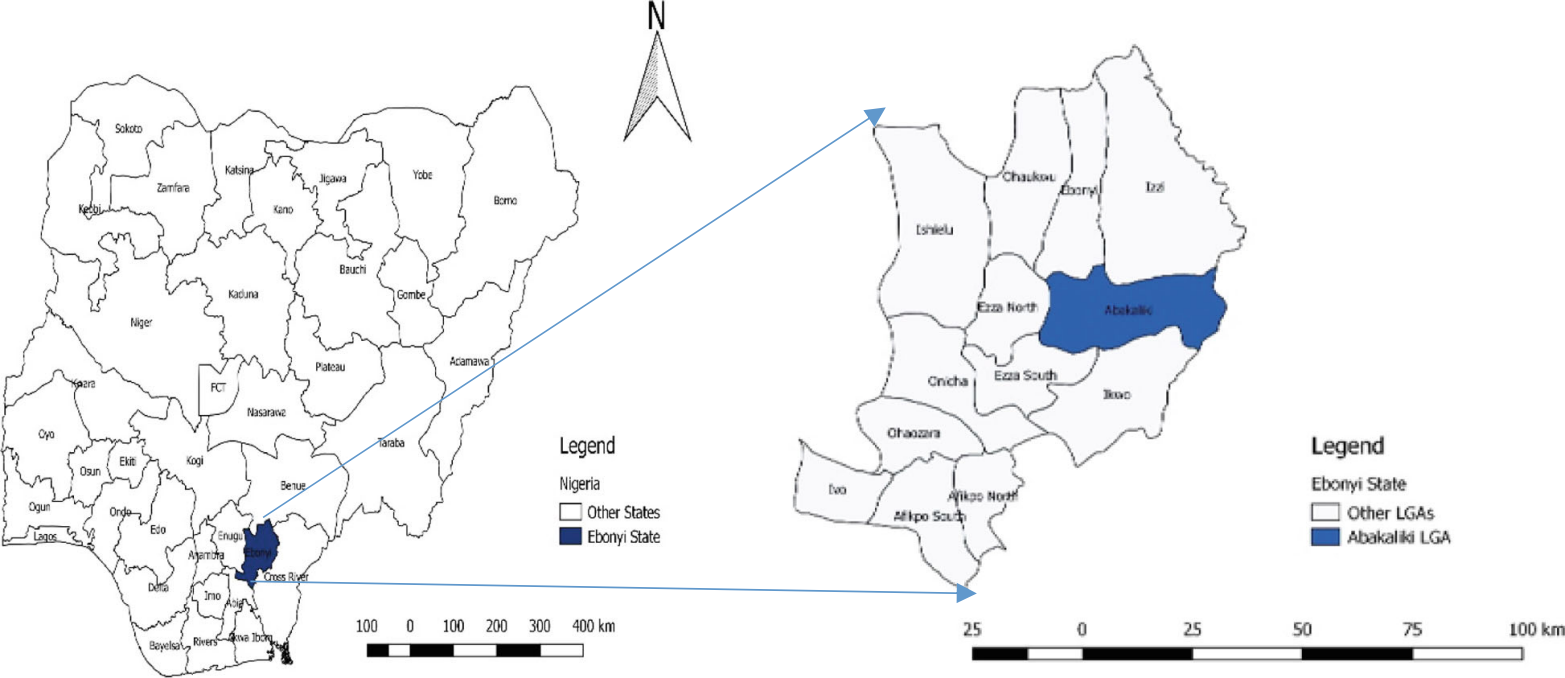
A map of Nigeria highlighting Ebonyi State and her LGAs. Developed using QGIS version 2.18.13 a free GIS software

We conducted a cross-sectional study in the LF affected wards in Abakaliki LGA of Ebonyi State, Nigeria. Abakaliki LGA is one of the 13 LGAs in Ebonyi state (See Fig. 1). It is one of the LGAs that make up the Abakaliki metropolis, the capital of the state. The LGA is made up of rural and urban areas with a projected population of 217,251 in 2019. It has a tropical climate with an average temperature of 27.7 °C and an average rainfall of 1918 mm. Their annual weather condition is made up of two distinct seasons, the wet (April to October) and dry season (November to March). The occupation of the people is diverse with a significant proportion of them being farmers (especially in rice, yam, cassava and palm kernel). The agricultural practices are influenced by seasons. Bush burning and open air drying on the floor are acceptable agricultural practices. The population comprises of predominantly Igbo and Christians. Abakaliki LGA is divided into 20 wards. During the 2019 LF outbreak in the state which started in Epi week 1, five of these wards were affected as at Epi week 8.

### Study population and sampling technique

The study was conducted among persons aged 18 years and above living in affected communities in Abakaliki LGA during the outbreak. We included all consenting adults who were present at homes at the time of the study while adults who were too sick to participate were excluded. Using the formula for the estimation of sample size for single independent proportion, $\Big(n=\frac{\ {\mathrm{Z}\upalpha}^2\mathrm{pq}}{\ {d}^2}$) [15], and the prevalence of good knowledge in an earlier study of 30% [16], Z_α_ of 1.96 at 95% confidence level and a precision of 5%, a minimum sample size of 323 was calculated. After correcting for 10% non-response, a sample size of 356 was used in the study.

We recruited 356 individuals who met our selection criteria, using a multistage sampling technique. In stage 1, three wards were selected from the list of five affected wards (Azungele, Egugwu, Ochiriozua, Onuofia, Ndiakparata, and Nkaliki) by balloting. The questionnaire was allocated to the selected wards proportionate to their size. In each of the selected wards two settlements were selected by balloting in Stage 2. Subsequently, two streets per settlement were selected using a table of random numbers in stage 3. On the streets, houses were selected using systematic random sampling technique. The sampling interval was obtained by dividing the number of households in the street by the sample size allocated to the street. In each household selected, only one eligible adult was interviewed, were there were more than one eligible adult, the list of all eligible adult was made and one was selected by simple random sampling through balloting. In the case of residual questionnaire per street, at the end of each selected street, the next street on the right was entered. The process was continued until sample size was met.

### Study tool

A pretested interviewer administered questionnaire was used. Each questionnaire consisted of 3 sections. Section A obtained data on the socio-demographic characteristics of the respondents, Section B obtained data on the knowledge of respondents about LF symptoms and risk factors while Section C obtained data on risk perceptions of the respondents using the HBM by Rosenstock. This questionnaire was developed by the authors specifically for the purpose of this study following a review of literature. See Additional file 1.

### Data collection

Twelve trained research assistants most of whom were involved in the state’s polio supplemental immunization campaign (house to house) and hence knowledgeable about the study area, administered the questionnaires. Field supervisors supported data collection daily by providing support to the research assistants and ensured the protocol was adhered to. Each street had a data collection team of at least 2 persons. Each team had at least one person who spoke and understood the native language. The questionnaires were translated orally into the local dialect by the interviewers for respondents who did not understand the English language. The research assistants were trained to ensure that participation in the study was voluntary and that confidentiality was maintained. Written informed consent was obtained from each respondent before data collection.

### Data management

The data was cleaned by checking for consistency and completeness. Data analysis was with Epi-Info version 7.2 and Microsoft Excel 2016.

Knowledge of LF infection was tested using 20 questions. Each correct response has a score of one with a maximum score of 20. Respondents who scored ≥70% of the maximum score were categorized as having good knowledge otherwise they were categorized as having poor knowledge [17].

Risk perception was assessed by examining respondents’ perceived susceptibility to LF infection, perceived severity of LF infection, perceived benefits of LF preventive practices, perceived barriers towards LF preventive practices and perceived self-efficacy towards LF preventive practices [18, 19].

Perceived severity was tested using 2 questions with a maximum score 10; perceived susceptibility was accessed using 2 questions with a maximum score 10; perceived benefit was accessed using 15 questions with a maximum score 75; perceived barrier was accessed using 1 question with a maximum score 5 while self -efficacy was accessed using 14 questions with a maximum score 70. A score of ≥80% of the maximum score was categorized as high perceived severity, susceptibility, benefit, barrier and self-efficacy respectively.

We calculated the proportions of respondents with good knowledge of LF infection, and high-risk perception. The relationship between the risk perception and sociodemographic characteristics was assessed using Chi square at 5% level of significance. The factors that had *p* value less than 0.2 were modelled in the multivariable logistic regression to determine the associated factors influencing their risk perceptions.

Risk perception (perceived severity, perceived susceptibility, perceived benefit, perceived barrier and perceived self-efficacy) were dependent variables.

Sociodemographic characteristics and knowledge level were independent variables.

## Results

A total of 356 respondents aged between 18 years and 80 years participated in the study. After data cleaning, only 329 questionnaires were entered for analysis. The mean age of the participants was 33.3 ± 12.2 years with the highest proportion of the respondents 124 (37.7%) being in the 25-34 year age group, females 208 (63.2%) and had secondary education 170 (51.7%) as their highest level. Their main occupation was trading (36.3%, Table 1).

**Table 1:** Socio-demographic Characteristics of Respondents in Abakaliki LGA, Ebonyi State, South Eastern Nigeria 2019

Variable	Frequency (*n* = 329)	Percentage (%)
Age group (years)		
15–24	82	24.9
25–34	124	37.7
35–44	66	20.1
45–54	32	9.7
> 54	25	7.6
Sex		
Female	208	63.2
Male	121	36.8
Marital Status		
Married	230	69.9
Single	98	29.8
Divorced	1	0.3
^a^Ethnicity(*n* = 325)		
Igbo	316	97.2
Hausa	6	1.9
Yoruba	2	0.6
Others	1	0.3
Education		
None	15	4.6
Primary	40	12.2
Secondary	170	51.7
Tertiary	104	31.6
^a^Religion(*n* = 323)		
Christianity	320	99.1
Islam	2	0.6
Traditionalist	1	0.3
^a^Occupation(*n* = 292)		
Traders	106	36.3
Civil Servant	51	17.5
Unemployed	50	17.1
Others	47	16.1
Farmer	38	13.0

^a^Missing values

**Fig. 2:**
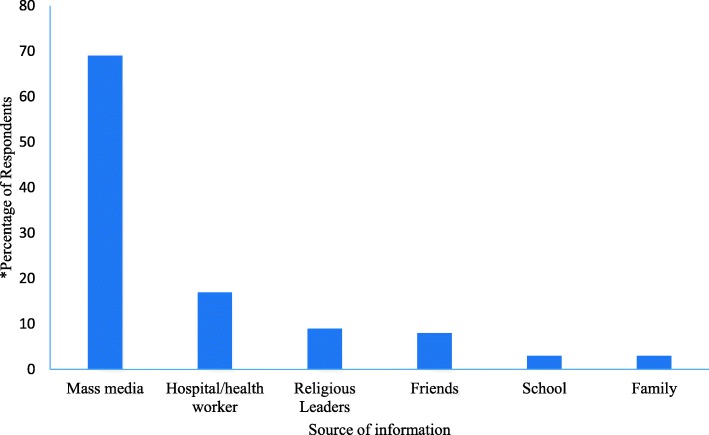
Participants’ source of Information among those who have heard of Lassa Fever in Abakaliki Local Government Area, Ebonyi State, South Eastern Nigeria 2019. *Multiple responses recorded

Almost all respondents had heard about LF infection 326 (99.1%). The main source of information on LF was mass media at 69% (Fig. 2). Out of those who had heard of LF and responded, 30.6% did not believe that LF was in their community and 19.2% did not know what to believe about the presence of LF in their community.

Among those who were aware of LF, 164 (50.3%) had poor knowledge of LF infection symptoms and risk factors. For further details on the knowledge of respondents on the symptoms and risk factors of LF see Table 2.

**Table 2:** Knowledge of Lassa fever symptoms and risk factors among respondents in Abakaliki Local Government Area, Ebonyi State, South eastern Nigeria 2019

Knowledge Variables (*n* = 326)	Yes (%)
Symptoms of Lassa Fever	
Fever	69.3
Bleeding	67.2
Body weakness	65.0
Headache	63.8
Vomiting	57.7
Chest pain	49.4
Sore throat	44.8
Diarrhoea	44.5
Cough	42.0
Facial swelling	41.7
Abortion/miscarriage in pregnant women	25.5
Risk factors	
Eating food contaminated by rats	88.7
Contact with rats, their feaces, blood or urine	83.1
Contact with someone sick with Lassa fever	76.7
Contact with dead body of Lassa fever victim	76.4
Rat bite	76.1
Dirty environment	73.9
Eating uncovered/unprotected food	73.6
Improper refuse disposal	69.3
Eating poorly cooked food	41.1

Concerning the risk perception, many [239 (73.3%)] thought that LF is a very serious infection and 134 (41.1%) believed they had a very large chance of contacting the infection if they didn’t take preventive measures against LF (Table 3). About getting LF infection, 78 (23.9%) of the participants were not concerned. One hundred and five (32.2%) were not likely to accept a person treated for LF in the community.

**Table 3:** Risk perceptions of Respondents in Abakaliki LGA, Ebonyi State, South Eastern Nigeria 2019

Risk Perception of Lassa Fever (*n* = 326)	Percentage (%)
Perception of Severity of Lassa Fever	
How Serious do you think Lassa Fever is?	
Very Serious	73.31
Serious	19.63
Neither Serious nor not Serious	0. 92
Slightly not Serious	3.37
Not serious at all	2.76
How would you feel if you were to contact LF next year?	
Very Serious	90.80
Serious	7.06
Neither Serious nor not Serious	1.23
Slightly not Serious	0.31
Not serious at all	0.61
Perception of Susceptibility to Lassa Fever	
Do you think that you can contact Lassa Fever in the future if you do not take any preventive measures?	
Certainly Yes	60.74
Probably Yes	22.70
Perhaps not, Perhaps yes	6.13
Probably not	4.91
Certainly not	5.52
What do you think are your chances of getting Lassa Fever in the future if you do not practice any preventive measure?	
Very Large Chance	41.10
Large Chance	29.75
Not small, not Large	10.74
Small Chance	12.27
Very small chance	6.13
Perception of Barrier towards preventive measures	
Are you concerned about contacting Lassa Fever?	
Very Concerned	37.73
Concerned	30.37
Slightly Concerned	7.98
Not Concerned	16.87
Not concerned at all	7.06
Perception of Benefit of preventive measures	
Is it necessary to carry out preventive measures against Lassa fever?	
Certainly Yes	74.23
Probably Yes	19.63
Perhaps not, Perhaps yes	4.29
Probably Not	1.53
Certainly Not	0.31

**Fig. 3:**
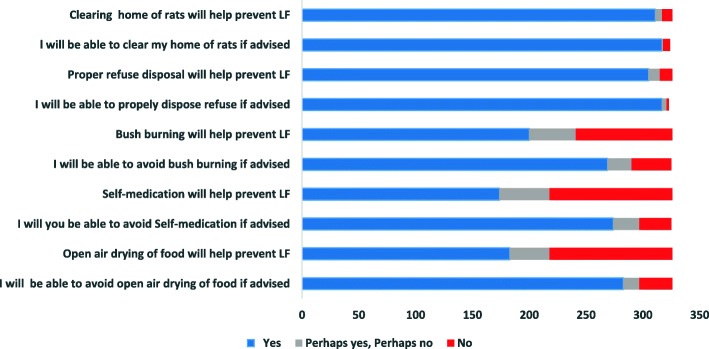
Perceived benefits of Lassa fever preventive practices and Self efficacy towards them by respondents in Abakaliki LGA, Ebonyi State, South Eastern Nigeria, 2019

Many participants believed that avoidance of direct contact with rats 219 (67.3%), stopping consumption of rats 231 (70.9%) and avoidance of contact with dead bodies of LF victims 252 (77.3%) will most certainly help prevent LF. Only 73 (22.4%) believed that contact with persons with LF infection will certainly not prevent spread of the infection. Self-medication [122 (37.4%)], bush burning [138 (42.3%)] and open-air drying [128 (39.3%)] were reported by the respondents to most certainly prevent the spread of the infection. A majority of the participants believed that they would most certainly be able to carry out LF preventive practices if advised. However, 29 (8.9%) and 35 (10.1%) said they won’t be able to avoid open air drying and bush burning if advised respectively (Fig. 3).

A large proportion of respondents (63.5%) had a low perceived benefit of LF preventive practices (Table 4). Good knowledge of LF infection and its risk factors was significantly associated with high perceived susceptibility towards LF infection (AOR:2.2, 95%CI: 1.25–3.3), high perceived severity of LF infection (COR:3.0, 95%CI:1.2–7.8) and high perceived benefit of LF preventive practices (COR:2.1,95%CI:1.3–3.3).

**Table 4:** Categorization of risk perceptions of respondents in Abakaliki LGA, Ebonyi State, South Eastern Nigeria 2019

Risk Perception of Participants	Frequency (*n* = 326)	Percentage (%)
Perception of severity of LF infection		
High	303	92.9
Low	23	7.1
Perception of susceptibility to LF infection		
High	236	72.4
Low	90	27.6
Perception of benefit of LF preventive practices		
High	119	36.5
Low	207	63.5
Perception of barrier towards LF preventive practices		
High	104	31.9
Low	222	68.1
Self-efficacy towards LF preventive practices		
High	269	82.5
Low	57	17.5

Male sex and younger persons (aged below 35 years) were associated with low perceived susceptibility towards LF infection, low perceived benefit and high perceived barrier towards LF preventive practices. However, none of these were statistically significant (Table 5).

**Table 5:** Factors influencing risk perception towards Lassa fever infection among residents of affected communities in Abakaliki LGA, South Eastern Nigeria 2019

Covariates	Crude OR (95%CI)	*P* value	Adjusted OR (95%CI)	*P* value
^a^High/Low Perceived Susceptibility				
Age Below 35 years	0.7 (0.4–1.1)	0.130	0.6 (0.3–1.0)	0.062
Male	0.7 (0.4–1.1)	0.168	0.6 (0.4–1.0)	0.053
Married	0.9 (0.5–1.6)	0.881	–	
Primary Education or less	1.6 (0.8–3.2)	0.293	–	
Good Knowledge	2.2 (1.4–3.7)	0.003	2.0 (1.25–3.3)	0.003
^a^High/Low Perceived Severity				
Age Below 35 years	1.1 (0.5–2.6)	1.000	–	
Male	1.4 (0.5–3.4)	0.665	–	
Married	0.8 (0.3–2.1)	0.845	–	
Primary Education or less	2.1 (0.5–9.4)	0.394	–	
Good Knowledge	3.0 (1.2–7.8)	0.003	–	
^a^High/Low Perceived Benefit				
Age Below 35 years	0.8 (0.5–1.3)	0.428	–	
Male	0.8 (0.5–1.2)	0.305	–	
Married	0.9 (0.6–1.5)	0.855	–	
Primary Education or less	1.0 (0.5–1.8)	1.000	–	
Good Knowledge	2.1 (1.3–3.3)	0.002	–	
^a^High/Low Perceived Barrier				
Age Below 35 years	1.3 (0.8–2.0)	0.446	–	
Male	1.2 (0.7–1.9)	0.585	–	
Married	1.2 (0.7–2.0)	0.648	–	
Primary Education or less	0.9 (0.5–1.7)	0.896	–	
Good Knowledge	0.9 (0.6–1.5)	0.779	–	
^a^High/Low Perceived Self Efficacy				
Age Below 35 years	1.4 (0.8–2.5)	0.313	–	
Male	0.7 (0.4–1.3)	0.288	–	
Married	0.6 (0.3–1.1)	0.141	–	
Primary Education or less	0.8 (0.4–1.6)	0.626	–	
Good Knowledge	1.2 (0.7–2.2)	0.595	–	

^a^Outcome variables with Low perceived susceptibility, low perceived severity, low perceived benefit, low perceived barrier and low perceived self -efficacy functioning as reference

## Discussion

In this study, we found that half of the respondents demonstrated a poor knowledge of LF fever symptoms and risk factors. This poor knowledge is worrisome despite the high level of awareness among the respondents. The high awareness of LF could be because of the high sensitization and awareness campaign about the infection during past epidemics in the state [20]. This finding was similar to a previous study by Nwonwu et al. in Izzi, Ebonyi state [16]. Olowoere et al. also reported a poor knowledge of LF by respondents in Ile Ife [21]. The finding of a high awareness of LF in this study, is similar to community-based studies in Lafia by Reuben and Gyar [22], and in Irrua by Ochei et al. [23] A low community awareness of LF in Owo, Ondo State has been reported by Ilesanmi et al. [24] and in Oshogbo by Adebimpe et al. [25]

Respondents in Abakaliki LGA had a better knowledge of LF risk factors compared to its symptoms suggesting a gap in risk communication information dissemination in the state and the need for risk communication content in the state to contain more LF symptoms for early identification of suspected cases. Fever, bleeding, body weakness and headache were the symptoms most correctly known by the respondents. Pregnancy loss was the least known symptom of LF. Eating of poorly cooked food was the least known risk factor for Lassa fever infection.

The media was the major source of information for Lassa fever in this study and it was similar to the findings of Aigbiremolen et al. [26] as well as Awosanya and colleagues [27]. This further emphasizes the important role of the media in disseminating information about Lassa fever to the public [20]; as the media has great potential to change the perception of its audience. In addition, respondents’ report of various sources of information for LF symptoms and risk factors (media, hospital/ Health care workers, family members, friends, school and religious leaders) point to the varied source of information for the infection. This has great import for the need to target all these groups with the right information on LF infection for correct LF information sharing within the state. The proportion of source of Lassa fever information from hospital/health care workers was low (17.7%). The fact that this was a community-based study involving healthy individuals could have biased the finding. However, there is a need for greater enlightenment of the public by the hospitals and health care personnel on Lassa fever when patients visit the hospitals irrespective on their diagnosis as a result of being resident in a Lassa fever endemic state.

Very high proportions of the respondents had a high perception of severity of LF infection, a high perception of susceptibility to LF infection if they don’t carry out preventive practices and a high perception of self-efficacy towards Lassa fever preventive practices. The high proportion of respondents who perceived LF as severe and who felt susceptible to LF infection could be a motivation for positive behavioural change (LF preventive practices) [9]. Sadly, the majority of respondents had a low perceived benefit of LF preventive practices. This could explain the reason why the LGA has had so many LF cases. In addition, LF prevention information fatigue among the respondents, as a result of recurrent LF epidemics despite LF prevention information from the media since 2012 when the first case was identified till date [20], may result in respondents having a lower perception of benefit of LF prevention practices. The presence of wrong perceived benefit of self-medication, bush burning and open air drying of food in preventing LF in some of the respondents is worrisome. This is because open air drying and bush burning are known practices that increase the risk of transmission of LFV as open air-drying exposes food to rodents which could lead to contamination with the LF virus while bush burning drives the movement of the rodents (multimammate rat) from the bushes into residents’ homes [1, 28]. Self-medication on the other hand can result in delayed presentation/identification of cases affecting treatment outcome as early administration of Ribavirin has been associated with a better treatment outcome confirmed LF cases [1, 29]. These wrong perceptions need to be targeted and corrected as well as safer agricultural practices in the risk communication content of the state.

A third of the respondents had a high perceived barrier towards LF preventive practices as these individuals were not concerned about getting LF. This could result in these individuals not carrying out preventive practices towards LF despite the available information on LF in the state. The high self-efficacy of the respondents towards LF preventive practices is commendable, signaling a great potential for the prevention and control of Lassa fever in the state. However, further sensitization is needed to help guide persons with low self-efficacy to adopt the right practices.

Good knowledge of LF and its risk factors was significantly associated with a high perception of severity of LF infection, a high perception of susceptibility of Lassa fever infection and a high perceived benefit of LF preventive practices. This relationship is similar to other studies that demonstrate a relationship between knowledge and the health belief constructs [30]. Good knowledge was also associated with high perception of self-efficacy and low barrier towards Lassa fever preventive practices but they were not statistically significant. Male sex and age below 35 years was associated with low perception of susceptibility but was not statistically significant. Persons with Secondary school education or more had a surprisingly low perception of severity though this was not significant. Educational level had no association with perception of benefit of Lassa fever preventive practices in this study.

A third of the respondents not likely to accept a person treated for Lassa fever was an additional finding in this study. This shows that there is some degree of stigmatization against persons with Lassa fever as reported in literature [22]. This could result in delayed presentation for treatment and a delay in case finding which is a crucial link in outbreak prevention and control.

This study was not without limitations. The restriction of this study to affected wards only was a limitation as a comparative study with unaffected wards in Abakaliki LGA would have given us a clearer understanding of the risk perceptions of inhabitants of Abakaliki LGA. The findings of this study may have been as a result of response bias of respondents to give positive answers they think the interviewer wants to hear. To mitigate this, respondents were advised on the importance of truthful response to the study and that wrong responses will not be penalized in any way. Recall bias in respondents was a potential limitation in this study. To maintain validity of the study, respondents were given the option of ‘I don’t know’ in the knowledge assessment options. In addition, the scarcity of risk perception studies on Lassa Fever served as a limitation to a robust comparison of findings from other studies on risk perception in LF. In spite of these, our study was strengthened by it being a community-based study and the in-dept analysis of the risk perception of residents of affected communities in Abakaliki LGA towards LF infection.

## Conclusions

In conclusion, the poor knowledge of LF among the respondents, and the high proportion of low perceived benefit of LF infection preventive practices shows a gap in the content and acceptance of LF risk communication information in the state despite the high level of perceived threat of LF and self-efficacy towards LF preventive practices. In addition, the significant relationship between good knowledge and perceived threat (perceived severity and perceived susceptibility) of LF infection and perceived benefit of LF preventive practices further buttresses the importance of good and accurate knowledge of LF on the perception which has great import on LF preventive practices. Therefore, there is a need to reexamine the risk communication content of the state towards LF in the community with special focus on the males and younger population as well as a need for a continuing education of the populace on the dangers of the disease at the community level.

We recommend a closer analysis of the LF risk communication content in the State (public and private). Sensitisation through media platforms should be all year round in the state with an added focus on the youth and male sex in addition to anti- LF stigma messages. We also recommend that the findings from this study serve as a guide to create Abakaliki LGA specific risk communication content towards LF infection prevention and control. In addition, we recommend a scale up of this study to the whole of Ebonyi State as well as a comparative study to compare the risk perception towards LF infection of residents living in affected and unaffected communities. We also recommend that residents should be further educated on the importance of avoiding bush burning and open air drying of their food.

## Supplementary information

**Additional file 1.** Questionnaire.

## Data Availability

All the data associated with this work is available from the corresponding author on reasonable request.

## Abbreviations

HBM: Health Belief Model
LF: Lassa fever
LGA: Local Government Area
OR: Odds Ratio
