# Supplementary material for: Knowledge and risk perception towards Lassa fever infection among residents of affected communities in Ebonyi State, Nigeria: implications for risk communication
Source: BMC Public Health. 2020 Feb 12;20:217. doi: 10.1186/s12889-020-8299-3 (PMC7017500; doi:10.1186/s12889-020-8299-3)
Supplement: Supplementary file 1 — Additional file 1. Questionnaire. [file 12889_2020_8299_MOESM1_ESM.docx]

**Risk perception to Lassa fever prevention and control study Questionnaire in Abakaliki Local Government, Ebonyi state**.

Good day Sir/ Ma, we will like to ask you some questions about your knowledge of Lassa fever as well as how you feel about its risk factors and prevention practices. You do not have to answer any question you are not comfortable with and can end the interview at any time. Your response is voluntary. Any information you give to us will be confidential. Please, if you have any question to ask about this survey, do not hesitate.

Please note that your participation in this survey implies that you have understood and have freely given your consent to participate in this study. You can withdraw this consent at any time.

Consent ………………

LGA ………………… Settlement …..………………..

WARD………………. Geo-cordinates: Longitude…….Latitude……..

Section A: Socio-demographics

1. Age in years (as at last birthday) …………….
2. Sex: 1. Male [ ] 2. Female [ ]
3. Marital Status: 1. Single [ ] 2. Married [ ] 3. Divorced [ ] 4. Separated [ ] 5. Others (Specify) ………………….
4. Ethnic group: 1. Hausa [ ] 2. Igbo [ ] 3.Yoruba [ ] 4.Others (specify) ……………..
5. Highest level of Education completed: 1. No formal Education [ ] 2. Primary [ ] 3. Junior Secondary [ ] 4. Senior Secondary [ ] 5.Tertiary [ ]
6. Occupation ………………………………………………….
7. Religion: 1. Christianity [ ] 2. Islam [ ] 3. Others (Specify) ………..

Section B: Knowledge of Lassa fever.

1. Have you ever heard of Lassa fever? 1. Yes [ ] 2. No [ ]
2. If yes, what is/are your source(s) of information? 1. Friends [ ] 2. Mass Media [ ] 3. Family [ ] 4. School [ ] 5. Hospital/ Health worker [ ] 6. Religious leaders [ ] 7. Others (specify) ………………….
3. Do you believe that Lassa fever is in your community? 1. Yes [ ] 2. No [ ] 3. I don’t know [ ]
4. What are the symptoms of Lassa fever?

| Symptoms | Yes | No | I don’t’ know |
| --- | --- | --- | --- |
| Fever |  |  |  |
| Headache |  |  |  |
| Sore throat |  |  |  |
| Diarrhoea |  |  |  |
| Vomiting |  |  |  |
| Bleeding |  |  |  |
| Body weakness |  |  |  |
| Cough |  |  |  |
| Chest pain |  |  |  |
| Facial swelling |  |  |  |
| Abortions/ Miscarriage in pregnant women |  |  |  |

1. How can people get Lassa fever?

| People can get Lassa fever by: | Yes | No | I don’t Know |
| --- | --- | --- | --- |
| Eating food contaminated by rat’s feaces and urine |  |  |  |
| By being bitten by rats |  |  |  |
| Contact with rats, their feaces, blood or urine |  |  |  |
| Improper refuse disposal |  |  |  |
| Dirty environment |  |  |  |
| Eating of uncovered/ unprotected stored food(cooked/uncooked) |  |  |  |
| Eating of poorly cooked food |  |  |  |
| Contact with someone sick with Lassa fever |  |  |  |
| Contact with dead body of someone who died from Lassa fever. |  |  |  |
| Others(Specify) |  | | |

Section C: Risk Perception.

1. How serious do you think Lassa fever is?

| Not serious at all | Slightly not serious | Neither serious nor not serious | Serious | Very serious |
| --- | --- | --- | --- | --- |
|  |  |  |  |  |

1. How would you feel if you were to contact the following diseases the next year?

| Disease | Not serious at all | Slightly not serious | Neither serious nor not serious | Serious | Very serious |
| --- | --- | --- | --- | --- | --- |
| Catarrh |  |  |  |  |  |
| Heart attack |  |  |  |  |  |
| Malaria |  |  |  |  |  |
| Diabetes |  |  |  |  |  |
| Stroke |  |  |  |  |  |
| Lassa Fever |  |  |  |  |  |
| Ebola |  |  |  |  |  |

1. Do you think that you can contract Lassa fever in future if you do not take any preventive measures?

| Certainly not | Probably not | Perhaps not, perhaps yes | Probably yes | Certainly Yes |
| --- | --- | --- | --- | --- |
|  |  |  |  |  |

1. What do you think are your chances of getting Lassa fever in the future if you do not practice any preventive measures?

| Very small chance | Small chance | Not small, not Large | Large chanced | Very large chance |
| --- | --- | --- | --- | --- |
|  |  |  |  |  |

1. Do you think it is necessary to carry out preventive measures against Lassa fever?

| Certainly not | Probably not | Perhaps not, perhaps yes | Probably yes | Certainly Yes |
| --- | --- | --- | --- | --- |
|  |  |  |  |  |

1. Are you concerned about contracting Lassa fever?

| Not at all concerned | Not concerned | Slightly concerned | Concerned | Very concerned |
| --- | --- | --- | --- | --- |
|  |  |  |  |  |

1. Are you willing to accept a person who has been treated for Lassa fever?

| Certainly not | Probably not | Perhaps not, perhaps yes | Probably yes | Certainly Yes |
| --- | --- | --- | --- | --- |
|  |  |  |  |  |

1. Do you think any of the following practices will help prevent Lassa fever?

|  | Certainly not | Probably not | Perhaps not, perhaps yes | Probably yes | Most certainly |
| --- | --- | --- | --- | --- | --- |
| Frequent Hand washing with soap and water |  |  |  |  |  |
| Stopping consumption of rats |  |  |  |  |  |
| Clearing home of rats |  |  |  |  |  |
| Avoiding direct contact with rats |  |  |  |  |  |
| Proper food storage (Rat resistant containers) |  |  |  |  |  |
| Proper refuse disposal |  |  |  |  |  |
| Proper sanitation at home |  |  |  |  |  |
| Proper environmental sanitation |  |  |  |  |  |
| Washing of fruits and vegetables before eating |  |  |  |  |  |
| Self-medication |  |  |  |  |  |
| Contact with persons with Lassa fever |  |  |  |  |  |
| Bush burning |  |  |  |  |  |
| Open air drying of food on the ground |  |  |  |  |  |
| Avoidance of contact with dead bodies of Lassa fever patients |  |  |  |  |  |

1. Do you think you will be able to do the following if advised?

|  | Certainly not | Probably not | Perhaps not, perhaps yes | Probably yes | Certainly Yes |
| --- | --- | --- | --- | --- | --- |
| Frequent Hand washing with soap and water |  |  |  |  |  |
| Stopping consumption of rats |  |  |  |  |  |
| Clearing home of rats |  |  |  |  |  |
| Avoiding direct contact with rats |  |  |  |  |  |
| Proper food storage (Rat resistant containers) |  |  |  |  |  |
| Proper refuse disposal |  |  |  |  |  |
| Avoidance of self medication |  |  |  |  |  |
| Proper sanitation at home |  |  |  |  |  |
| Proper environmental sanitation |  |  |  |  |  |
| Washing of fruits and vegetables before eating |  |  |  |  |  |
| Avoidance of contact with persons with Lassa fever |  |  |  |  |  |
| Avoidance of Bush burning |  |  |  |  |  |
| Avoidance of open air drying of food on the ground |  |  |  |  |  |
| Avoidance of contact with dead bodies of Lassa fever patients |  |  |  |  |  |

Thank you for your time.
